# O armário: Fruiting phenology data for 4,462 plant taxa in Portugal (1926–2013)

**DOI:** 10.1038/s41597-024-03520-9

**Published:** 2024-06-22

**Authors:** Ruben Heleno, José M. Costa, Filipe Covelo, Joaquim Santos, Pedro Lopes, António C. Gouveia, Arménio Matos, Agostinho Salgado, M. Teresa Girão da Cruz, João Farminhão, Marta Horta, Guilherme Barreto, Ana V. Marques, Leonardo Craveiro, Patrícia Pinto, Matilde Santos, Bárbara Nunes, Margarida Barreiro, André Dias, Gabriel Rodrigues, Leonor Esteves, Marina Wanderley, Inês Santos, José Pedro Artiaga, João Veríssimo, Inês Vilhena, Lucas Moniz, Arthur Leão, Marta Couras, Sara B. Mendes, Mauro Nereu, Ana Margarida Dias da Silva, Fátima Sales, M. Teresa Gonçalves, António Coutinho, Helena Freitas, Joaquim S. Silva, Jaime Ramos, Elizabete Marchante, Sérgio Timóteo

**Affiliations:** 1 Centre for Functional Ecology, Associate Laboratory TERRA, Department of Life Sciences, University of Coimbra, Calçada Martim de Freitas, 3000-456 Coimbra, Portugal; 2 Botanic Garden of the University of Coimbra, University of Coimbra, Calçada Martim de Freitas, 3000-456 Coimbra, Portugal; 3 IATV - Instituto do Ambiente, Tecnologia e Vida, Rua Sílvio Lima, Pólo II, 3030-790 Coimbra, Portugal; 4 Escola Superior Agrária, Instituto Politécnico de Coimbra. Bencanta, 3045-601 Coimbra, Portugal; 5 Centro de História da Sociedade e da Cultura, University of Coimbra, 3000-370 Coimbra, Portugal; 6 MARE - Marine and Environmental Sciences Centre/ARNET – Aquatic Research Network, Department of Life Sciences, University of Coimbra, Calçada Martim de Freitas, 3004-517 Coimbra, Portugal

**Keywords:** Plant ecology, Plant reproduction

## Abstract

Species phenology - the timing of key life events - is being altered by ongoing climate changes with yet underappreciated consequences for ecosystem stability. While flowering is generally occurring earlier, we know much less about other key processes such as the time of fruit ripening, largely due to the lack of comprehensive long-term datasets. Here we provide information on the exact date and site where seeds of 4,462 taxa were collected for the *Index Seminum* (seed exchange catalogue) of the Botanic Garden of the University of Coimbra, between 1926 and 2013. Seeds were collected from spontaneous and cultivated individuals across Portugal, including both native and introduced taxa. The database consists of 127,747 curated records with information on the species, or infraspecific taxa (including authority), and the day and site where seeds were collected. All records are georeferenced and provided with a confidence interval for the collection site. Taxonomy was first curated manually by in-house botanists and then harmonized according to the GBIF backbone taxonomy.

## Background & Summary

There is clear evidence that ongoing climate change is rapidly altering the timing of key recurring life events – species phenology – including plant flowering, insect emergence, or bird migration^1–3^. Indeed, phenological shifts are one of the first responses of organisms to environmental changes and thus one of the more sensitive biological indicators of climate changes, largely preceding other more insidious responses such as range shifts or extinctions^4,5^. The growing realization of the importance of phenology on ecosystem functioning and stability has triggered a revival of phenological research in recent decades, spearheaded by research on flowering phenology^6–8^.

While flowering is key for pollination and plant reproduction, the production of seeds and fruits is at least as important, for it is only during this short period that plants can colonize new sites or endure periods of unfavourable environmental conditions through seed dormancy^9,10^. Indeed, the timeframe available for fruit production is a key driver of global diversity patterns and is central to understand how these can be affected by climate change^11^. Furthermore, evidence shows that the drivers of fruit ripening are not necessarily the same as those driving flowering phenology^12–14^, rendering fruiting phenology research particularly needed^14,15^. Fruiting phenology has important ecological and conservation implications, as reviewed in Morellato *et al*.^16^, including the potential to create mismatches between the availability of ripe fruit and their migratory seed dispersers^17,18^, modulating the dispersal services available to invasive alien plant species^19,20^, or determining regeneration potential after wildfires^21,22^. All of these have a recognized potential to change the composition of future ecosystems, especially forests^15,18,23^. It is thus unfortunate that fruiting season information is generally not available from botanical species descriptions in the same way that flowering is.

Fruiting phenology information can be obtained by several methods. The most straightforward is the establishment of long-term phenological stations where plants are periodically inspected (ideally daily) and the date of the first ripe fruit on multiple individuals is recorded^24^. Alternatively, fruiting can also be identified by periodically checking fruit traps^25^. However, while these methods originated some of the most comprehensive and accurate datasets on fruiting phenology available to date, they require a very large commitment in terms of continued sampling effort, particularly challenging under the constraints of short funding cycles, and therefore not practical to characterize entire floras over long temporal series and large spatial scales. The compilation of metadata from biological collections, chiefly from herbarium specimens, has been a highly valuable solution e.g.^8,26^. However, this approach also comes with its own intrinsic biases^27,28^ and is particularly suited to track flowering phenology due to the taxonomic value of flowers, more commonly present in herbarium specimens than fruits^29^.

Although fruiting phenology studies are not uncommon, their taxonomic coverage and duration is generally low^30^. In particular, due to stringent trade-offs between the number of species included and effort required to monitor them^31^, it is possible to find some remarkably long-term datasets e.g. a single species followed for 633 years^32^, and some remarkably comprehensive studies e.g. 1202 species followed for 7 years^33^. However, to our knowledge, no study to date has managed to follow any sizeable fraction of an entire flora for more than a decade^15^. While new technological solutions, such as artificial intelligence and large-scale citizen science initiatives, can facilitate the automated collection of massive contemporaneous data^16^, they cannot offer solutions to reconstruct past phenology against which recent shifts can be compared^28^.

Here we explore the historical dataset of a longstanding seed exchange program that has documented fruiting phenology data for a broad spectrum of species over an extensive temporal series. This dataset was made possible by the renewed interest on the natural sciences and the proliferation of botanical gardens in the late 18^th^ century, when some gardens established seed and plant exchange programs to expand and preserve their botanical collections and to resolve taxonomical ambiguities^34^. To facilitate this exchange, numerous Botanical Gardens published a list of seed species available yearly, known as *Index Seminum* (Latin for: Seed Catalogue), many continuing to be issued to this day^35^. The *Index Seminum* of the Botanic Garden of the University of Coimbra started in 1868 and was considerably improved in 1926 by expanding and diversifying taxa and collection range, and standardizing identification, storage and distribution of seeds^36^. Most importantly, there were also improvements in the gathering and storage of the information associated to each collected seed, which started to include the name of the species, subspecies, variety or form of the plants, taxonomic authority, as well as the exact collection date and site. By 1932, the Botanic Garden was regularly exchanging seeds with 359 institutions worldwide, and at its peak, the service offered seeds of 2,758 species, shipping over 11,000 seed packages to 800 scientific institutions around the globe^37,38^.

## Methods

**Fig. 1:**
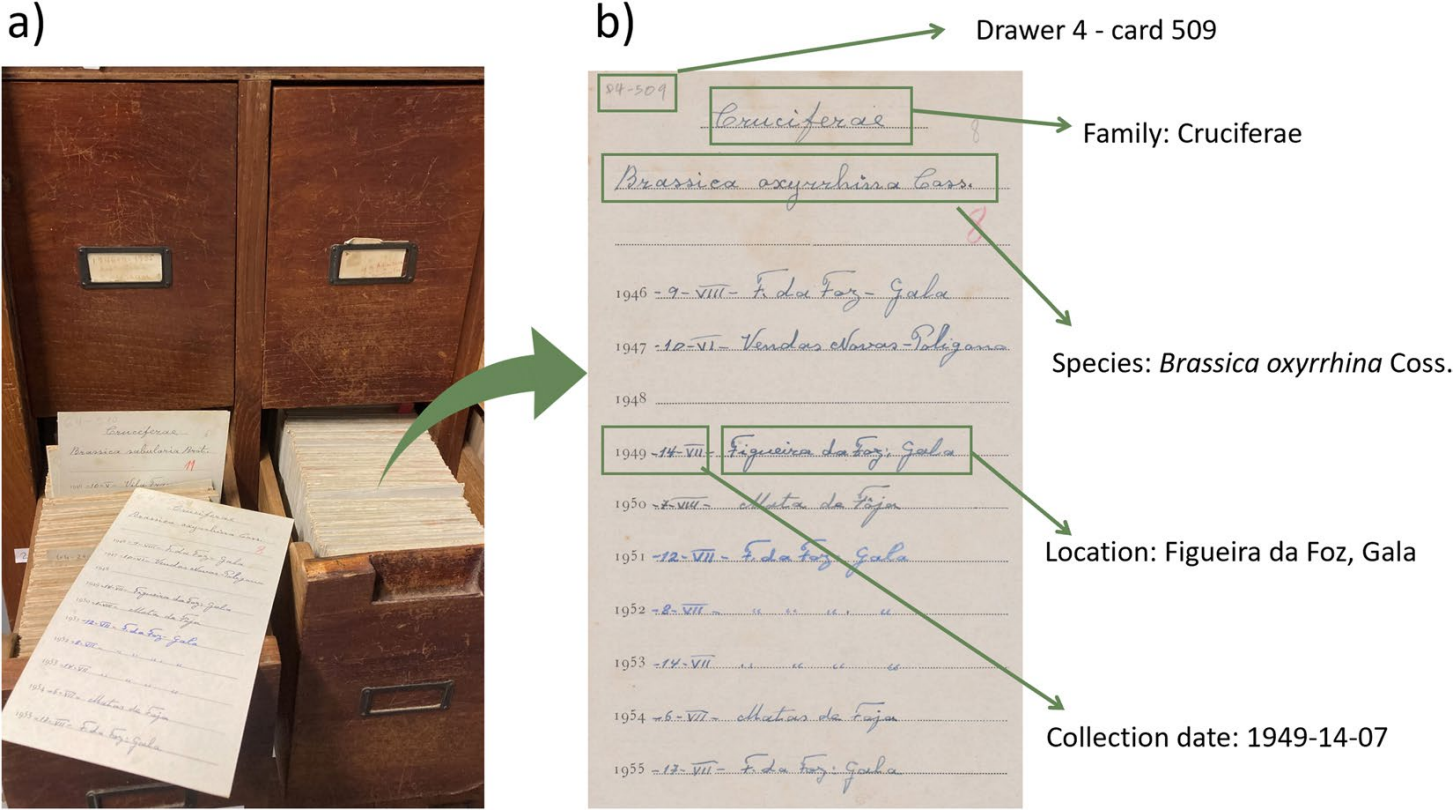
General aspect of the original data support. (**a**) detail of the storage cabinet showing 4 drawers containing the data recording cards for each species; (**b**) example of one out of the 23,006 cards from where the original data was extracted.

**Fig. 2:**
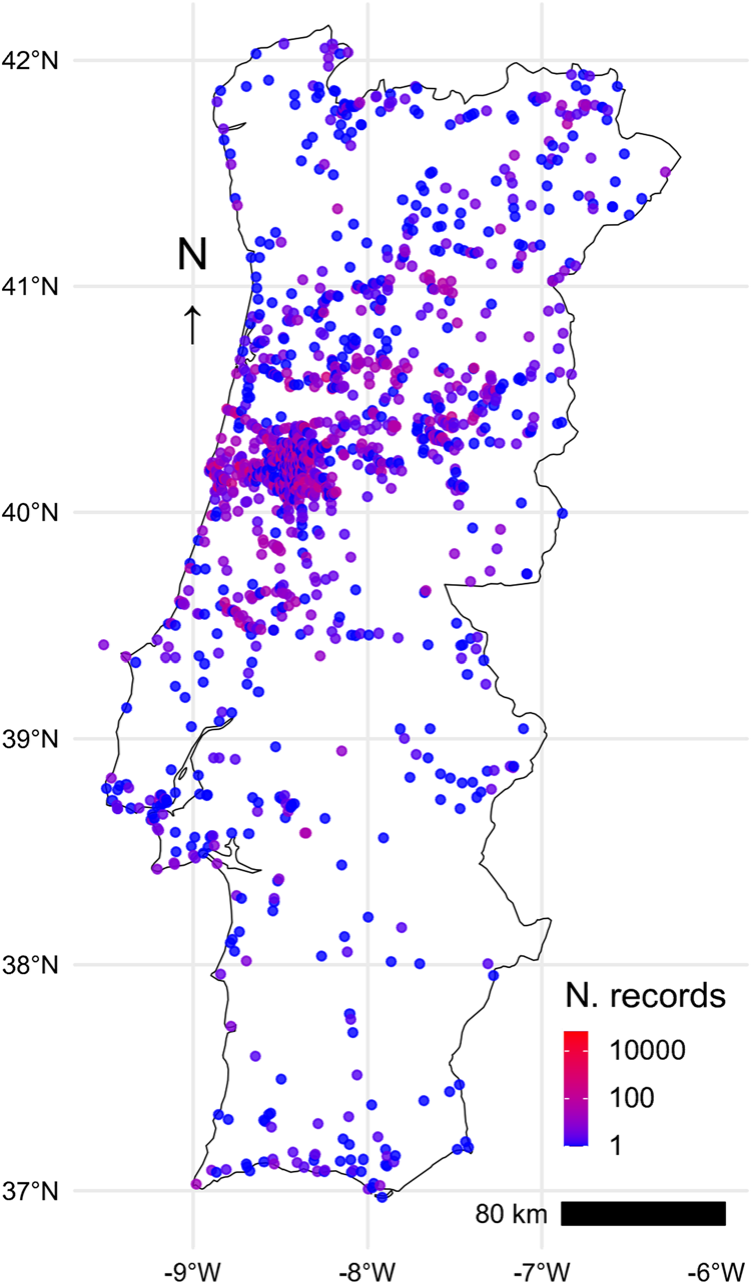
Spatial distribution of the 127,747 records included in the database.

Our dataset includes the records collected since 1926 by the staff of the Botanic Garden of the University of Coimbra that include the date, location, and species or infraspecific taxa for the seeds collected every year to integrate the seed exchange catalogue. These records were stored in a wood cabinet (“armário” in Portuguese) and kept in the original handwritten cards, to which every year a new location and date was added when each taxon was newly collected (Fig. 1). The dataset includes both native and introduced species, as well as spontaneous and cultivated species collected inside the Botanic Garden, but also on dedicated field trips across continental Portugal, including the Berlengas island (Fig. 2). The initial dataset included 138,191 entries, which were carefully curated and georeferenced, resulting in 127,747 fully validated records after discarding incomplete, dubious or duplicated records, as well as those referring to reproductive organs other than seeds (i.e. bulbs and fern spores). Finally, a small proportion of the most recent records (2.7%) were retrieved directly from field notebooks that had not been incorporated into the cards catalogue, and an additional 0.9% were retrieved from the online catalogue of the Herbarium of the University of Coimbra, where they have been directly entered (https://coicatalogue.uc.pt, accessed on 2023-01-05). The complete dataset includes collection records for 4,462 plant taxa.

The day of collection indicates that at least one individual plant of that taxon was fruiting on a given day, at a given site. Since the collected seeds were destined to germplasm exchange programs, collectors specifically targeted ripe fruits with viable seeds. This means that seeds that were not fully formed and likely to be viable (based on the accumulated experience of the collectors/gardeners for each plant species) would not be collected and that site would need to be revisited latter to collect ripe fruits.

### Taxonomic harmonization

Botanical nomenclature was first manually verified by in-house botanists that uniformized small spelling mistakes and confirmed the taxonomic authorities. This consolidated list was then harmonized with the Global Names Resolver with function *gnr_resolve()* in R^39^, with the package *taxize 0.9.9*^40,41^, against the Global Biodiversity Information Facility (GBIF) backbone taxonomy accessed on 2023-03-01. The accepted taxon name and taxonomic rank were extracted at this stage (Table 1).

**Table 1:** Description of the field terms used in the database according to the Darwin Core guidelines^52^.

Term	Description
catalogNumber	Unique record number within dataset
occurrenceID	Persistent global unique identifier number, generated by https://guidgenerator.com/
otherCatalogNumbers	Number of the drawer and card (in the example: Drawer 15, card 314; see also Fig. 1b)
basisOfRecord	Type of data recording method
year	Year of collection
month	Month of collection
day	Day of collection
eventDate	Date of collection in the format yyyy-mm-dd
dynamicProperties	Complete name and authority of the taxon accepted in WCVP^42^. Formatted in JSON.
scientificName	Complete name and authority of the taxon accepted in GBIF^50^.
class	Taxonomic class of the taxon (GBIF)
order	Taxonomic order of the taxon (GBIF)
family	Taxonomic family of the taxon (GBIF)
genus	Taxonomic genus of the taxon (GBIF)
establishmentMeans	Native or Introduced taxon in Portugal, according to the WCVP
verbatimIdentification	Original taxon used in automated homogenization following curation by in-house botanists
locality	Lowest unified toponymy of the collection site
decimalLatitude	Latitude (in decimal degree) of the site where the plant was fruiting
decimalLongitude	Longitude (in decimal degree) of the site where the plant was fruiting
coordinateUncertaintyInMeters	Coordinate uncertainty in meters
minimumElevationInMeters	Elevation (m) above sea level, obtained with the Google Elevation API from coordinates
continent	Continent of the collection site
country	Country of the collection site
stateProvince	First order administrative region (”*Distritos*”), equivalent to states or provinces, from Google Geocoding API
municipality	Second order administrative region (“*Concelhos*”) equivalent to municipalities, obtained by Google Geocoding API
locationRemarks	Whether the seeds were collected inside or outside the Botanic Garden
verbatimLocality	Original location name written on the card with a minimum level of curation
georeferencedBy	Person responsible for georeferencing the collection site and associated uncertainty
geodeticDatum	Geodetic datum upon which the geographic coordinates are based
georeferenceProtocol	Manual used to determine the geographic coordinates and uncertainties
link_radius_map(only available on figshare)	Direct link for the unified location of each collection locality.
Binomial (only available on figshare)	Name of the accepted Genus and specific epithet of each record without the authority. This is not a Darwin Core field, but it’s useful for most common data manipulations
licence	Licence associated with the record

The list of native species for Portugal was extracted from the World Checklist of Vascular Plants (WCVP^42^) with function *wcvp_distribution()* in the R package *rWCVP 1.2.4*^43^ accessed on 2023-06-28. Species that were collected in the country but are not considered native were classified as introduced. To facilitate data interoperability, the dataset includes the original name, as well as the harmonized taxonomy according to both GBIF and the WCVP.

### Georeferencing protocol

Throughout the 87 years of data collection, the same collection site was often recorded with slightly different wording by different generations of collectors. The original list of localities, containing 3,753 distinct entries, was initially clustered based on the textual description using OpenRefine and then manually confirmed and further grouped into 1,485 unique curated localities. This clustering was performed only on toponymical homogenization without any loss of spatial accuracy (i.e., all unique sites were preserved and not clustered into broader categories). The final list of localities was georeferenced using the point-radius georeferencing method^44,45^. The latitude and longitude of each point and the confidence level for each coordinate was obtained using the online tool available on Maps.ie^46^ and coordinate uncertainty was calculated according to the Georeferencing Calculator^47,48^. The administrative levels below country (stateProvince and municipality) were obtained from the Google Geocoding API^49^ by submitting the latitude and longitude coordinates to the Reverse Geocoding Service. The estimated altitude for each pair of coordinates (minimumElevationInMeters) was obtained using the Google Elevation API.

## Data Records

The dataset is available at GBIF^50^ as a species occurrences map, and can also be downloaded from figshare^51^ as a single text file with information on 127,747 records arranged along 33 columns (total file size 89MB). Table headings follow the Darwin Core guidelines^52^.

## Technical Validation

The work largely benefited from the experience of Arménio Matos, Agostinho Salgado, and António Coutinho who actively participated in field sampling campaigns since 1972 and were thus familiarized with the collection protocols, species, and collection sites. The accumulated knowledge of the Herbarium of the University of Coimbra (COI) staff, namely Filipe Covelo, Joaquim Santos, and Fátima Sales, was also invaluable in curating the dataset, as many seeds were collected from the same populations (and often collected simultaneously from the same individuals) from where herbarium specimens were also collected.

### Final quality check

Intermediate quality checks were routinely performed during data entry, taxonomic harmonization and georeferencing to detect and correct errors. Lastly, when the dataset was completed, we performed a new and standardized quality check to evaluate the accuracy of the data. For this, we randomly selected 1,000 records using a random number generator and carefully rechecked all the information against the original cards. We found data transcription errors on 7 records that resulted in errors on the collection day (n = 3 records), month (n = 3 records), and taxa (n = 1 record), corresponding to an overall error rate of 0.7%.

## Usage Notes

The names provided in the fields “ScientificName” have already been harmonized according to the GBIF Backbone Taxonomy (see *Taxonomic harmonization* above). Therefore, for future taxonomical clarifications, users should use the “verbetimIdentification” field which corresponds to the original taxonomic treatment with only minor in-house manual corrections. To facilitate data interoperability, species names according to the WCVP is also provided in the subfield “scientificName_WCVP” in “dynamicProperties .

**Fig. 3:**
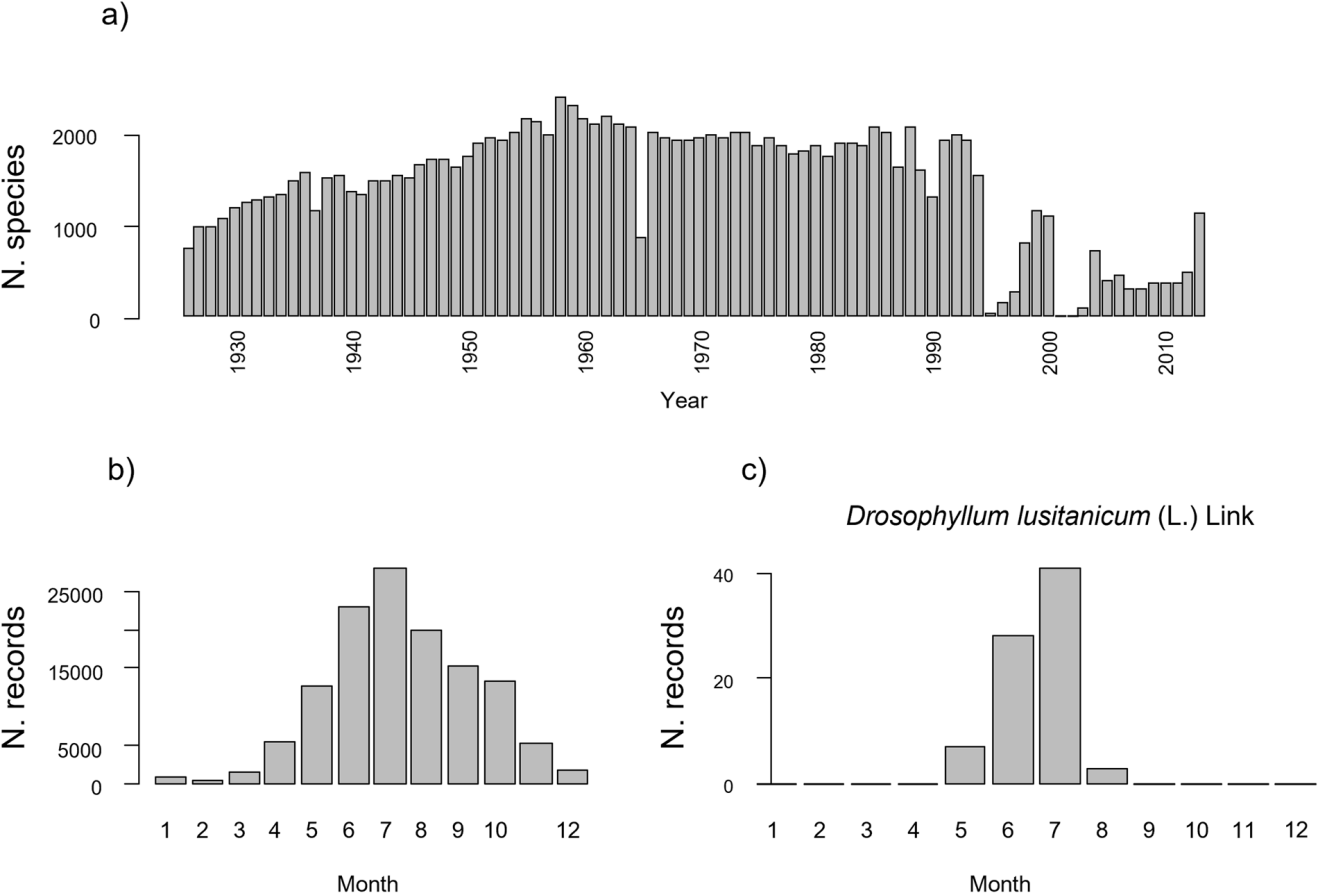
Basic data diagnostic plots. (**a**) Number of species collected each year; (**b**) Number of records per month, corresponding to the overall fruiting phenology of all species combined; (**c**) Example of the fruiting phenology (i.e. number of records) per month for a single focal species.

## Data Availability

The following code can be used to import the dataset into R and to produce basic descriptive plots at the general and specific levels (Fig. 3). Since some taxonomic authorities (i.e. the name of the taxon author) include apostrophes, such as *Tetragonia crystallina* L’Hér., to import data correctly, it might be necessary to specify that these are not quotes (with, quote = "\"",). data < - read.csv("~path/data.csv", quote = "\"", sep = ";") #Number of unique species collected per year (Fig. 3a) barplot (rowSums(table(data$year, data$scientificName) >0)) # Number of records on the entire dataset per month (Fig. 3b) barplot (table(data$month)) # Number of records per month for a single species (Fig. 3c) with(subset(data, scientificName == "Drosophyllum lusitanicum (L.) Link"),barplot(table(factor(month, levels = 1:12)), xlim = c(1, 12))).
